# Inter-Ethnic Differences in Quantified Coronary Artery Disease Severity and All-Cause Mortality among Dutch and Singaporean Percutaneous Coronary Intervention Patients

**DOI:** 10.1371/journal.pone.0131977

**Published:** 2015-07-06

**Authors:** Crystel M. Gijsberts, Aruni Seneviratna, Imo E. Hoefer, Pierfrancesco Agostoni, Saskia Z. H. Rittersma, Gerard Pasterkamp, Mikael Hartman, Leonardo Pinto de Carvalho, A. Mark Richards, Folkert W. Asselbergs, Dominique P. V. de Kleijn, Mark Y. Chan

**Affiliations:** 1 Laboratory of Experimental Cardiology, University Medical Center Utrecht, Utrecht, the Netherlands; 2 The Netherlands Heart Institute (ICIN), Utrecht, the Netherlands; 3 Cardiac Department, National University Hospital, Singapore; 4 Department of Cardiology, University Medical Center Utrecht, Utrecht, the Netherlands; 5 Department of Surgery, Yong Loo Lin School of Medicine, National University of Singapore, Singapore; 6 Saw Swee Hock School of Public Health, National University of Singapore, Singapore; 7 Department of Cardiology, Albert Einstein Hospital, Sao Paulo, Brazil; 8 Cardiovascular Research Institute (CVRI), National University Heart Centre (NUHCS), National University Health System, Singapore; 9 Durrer Center for Cardiogenetic Research, ICIN-Netherlands Heart Institute, Utrecht, The Netherlands; 10 Institute of Cardiovascular Science, faculty of Population Health Sciences, University College London, London, United Kingdom; Children's National Medical Center, UNITED STATES

## Abstract

**Background:**

Coronary artery disease (CAD) is a global problem with increasing incidence in Asia. Prior studies reported inter-ethnic differences in the prevalence of CAD rather than the severity of CAD. The angiographic “synergy between percutaneous coronary intervention (PCI) with taxus and cardiac surgery” (SYNTAX) score quantifies CAD severity and predicts outcomes. We studied CAD severity and all-cause mortality in four globally populous ethnic groups: Caucasians, Chinese, Indians and Malays.

**Methods:**

We quantified SYNTAX scores of 1,000 multi-ethnic patients undergoing PCI in two tertiary hospitals in the Netherlands (Caucasians) and Singapore (Chinese, Indians and Malays). Within each ethnicity we studied 150 patients with stable CAD and 100 with ST-elevated myocardial infarction (STEMI). We made inter-ethnic comparisons of SYNTAX scores and all-cause mortality.

**Results:**

Despite having a younger age (mean age Indians: 56.8 and Malays: 57.7 vs. Caucasians: 63.7 years), multivariable adjusted SYNTAX scores were significantly higher in Indians and Malays than Caucasians with stable CAD: 13.4 [11.9-14.9] and 13.4 [12.0-14.8] vs. 9.4 [8.1-10.8], p<0.001. Among STEMI patients, SYNTAX scores were highest in Chinese and Malays: 17.7 [15.9-19.5] and 18.8 [17.1-20.6] vs. 15.5 [13.5-17.4] and 12.7 [10.9-14.6] in Indians and Caucasians, p<0.001.

Over a median follow-up of 709 days, 67 deaths (stable CAD: 37, STEMI: 30) occurred. Among STEMI patients, the SYNTAX score independently predicted all-cause mortality: HR 2.5 [1.7-3.8], p<0.001 for every 10-point increase. All-cause mortality was higher in Indian and Malay STEMI patients than Caucasians, independent of SYNTAX score (adjusted HR 7.2 [1.5-34.7], p=0.01 and 5.8 [1.2-27.2], p=0.02).

**Conclusion:**

Among stable CAD and STEMI patients requiring PCI, CAD is more severe in Indians and Malays than in Caucasians, despite having a younger age. Moreover, Indian and Malay STEMI patients had a greater adjusted risk of all-cause mortality than Caucasians, independent of SYNTAX score.

## Background

Inter-ethnic differences in the prevalence of coronary artery disease (CAD) and cardiovascular risk factors such as diabetes [1] and dyslipidemia [2] are known. People of Indian (or South Asian) descent have been reported to have an unfavorable risk factor profile (e.g. higher prevalence of diabetes and dyslipidemia [3,4]) and a higher prevalence of CAD (as reported by the World Health Organization [5]) compared with Caucasians. Individuals of Chinese descent, on the other hand, have been reported to have a more favorable risk factor profile (e.g. low C-reactive protein levels [2] and low insulin levels [6]) and lower prevalence of CAD (as assessed by coronary artery calcium (CAC) scoring).[7]

The World Health Organization has projected that the majority of the global population of patients with CAD will be of Asian descent by 2030.[5] Yet, data on differences in the CAD burden among the individual Asian ethnic groups are sparse and predominantly based on Western (European) literature on Asian immigrants.[8] As such, the American Heart Association has assigned a high priority to multi-ethnic research on the burden and outcomes of CAD.[9]

Studies assessing CAC scores have shown that CAC scores are higher among community-dwelling individuals of Indian descent as compared with those of Chinese descent.[6,10,11] But, despite its sensitivity in detecting CAD, CAC scoring remains a screening tool that has limited specificity for the presence of underlying CAD. Coronary angiography remains the gold standard for assessing the presence and severity of CAD. Angiographic studies quantifying the severity of CAD are sparse; one study compared mainland Chinese with Australian Caucasians, showing less severe CAD in Chinese than in Caucasian coronary angiography patients as quantified by the Gensini score.[12]

In the context of significant multi-vessel CAD the angiographic synergy between percutaneous coronary intervention (PCI) with taxus and cardiac surgery (SYNTAX) score has been developed.[13] This score quantifies the anatomic extent and complexity of CAD over 16 anatomically defined coronary segments on coronary angiography. The SYNTAX score has been validated for predicting outcomes of patients undergoing PCI.[14] Based on the available literature on inter-ethnic differences in risk factor burden and CAD prevalence, we hypothesized that the severity of angiographic CAD, as quantitatively measured by SYNTAX score, differs among Caucasians, Chinese, Indians and Malays, who constitute four of the largest ethnic groups in the world [15]. For this purpose we investigated PCI patients from two tertiary hospitals: the University Medical Center Utrecht, the Netherlands (enrolling Caucasian patients) and the National University Hospital, Singapore (enrolling Chinese, Indian and Malay patients). In two well-circumscribed cardiologic patient groups: stable CAD and STEMI patients undergoing PCI, we investigated inter-ethnic differences in the severity of angiographic CAD by means of the SYNTAX score. Furthermore, we evaluated inter-ethnic differences in all-cause mortality, adjusted for SYNTAX score.

## Methods

### Study population

Patients were retrospectively, consecutively selected from the coronary angiography databases of two hospitals: the University Medical Center Utrecht (UMCU) in the Netherlands and the National University Hospital (NUH) in Singapore between December 2007 and October 2013. From the two sites, four ethnic groups were included in this study: Caucasians from the UMCU and Chinese, Indians and Malays from the NUH.

From a power calculation on a preliminary cohort of 20 stable CAD and 20 STEMI patients per ethnic group we concluded that 150 stable CAD and 100 STEMI patients per ethnic group were needed for the current study in order to give us 80% power to detect a difference in SYNTAX score of 2.4–5.7 points (SD 1.5–1.6) between ethnic groups, considering a Bonferroni post-hoc α of 0.0125.

For the current study, we selected 150 patients from each ethnic group who were diagnosed with stable CAD, defined as stable angina or angina equivalent, exertional dyspnea relieved either by rest or by nitroglycerin or silent myocardial ischemia.[16] Only patients with stable CAD requiring PCI as a treatment [16] were selected for the current study. Additionally, we selected 100 patients from each ethnic group with a clinical diagnosis of ST-elevated myocardial infarction (STEMI) [17] and who underwent primary PCI. We selected patients from October 2013 backwards and went as far back as necessary (December 2007), to allow us to obtain the desired number of patients. Patients with a history of coronary artery bypass grafting (CABG) surgery were excluded, as the regular SYNTAX score [13] which was used in this study is only applicable to the native coronary system.

### Ethics statement

The institutional review boards of both participating hospitals exempted this retrospective database study from approval. The exempts are registered at the NUH (Domain Specific Review Board, DSRB) under: 2012/00971 and at the UMCU (Medical Ethics Committee, METC) under: 13/222. This study conforms to the declaration of Helsinki.

No informed consent was necessary as data were analyzed anonymously.

### Ethnicity documentation

In Singapore, trained staff recorded self-reported ethnicity as documented on state-issued identification cards using one of the following categories: Chinese, Malay, Indian and other. All Dutch patients were assumed to be Caucasian for this study (from a questionnaire among a group of 1,429 angiography study patients >94% were confirmed as Caucasian and only 2% were of Asian descent).

### Risk factors

Diabetes was defined as any type of diabetes (fasting glucose >7mmol/L) [18] in the medical history or during index admission requiring medical treatment by means of oral glucose regulating medication or insulin injections (impaired glucose tolerance is not considered to be diabetes in this study). Hypertension is considered when mentioned in the patient’s medical history or when diagnosed during the index admission (systolic blood pressure >140 mmHg or diastolic blood pressure >90 mmHg) and/or the use of one or more antihypertensiva.[19]

Dyslipidemia was defined by any dyslipidemia requiring treatment in the medical history or during index admission as recommended by the ESC/EAS [20] guidelines. Smoking status was divided into three groups; current smoker, ex smoker (> 1 year since last smoke) and non-smoker. Advanced renal failure was defined as any renal disease requiring treatment with oral medication (phosphate-binding medications) or any type of renal replacement therapy in the medical history.[21]

Body mass index (BMI) was calculated by dividing weight (in kilograms) by squared height (in meters).

### Medication use

The use of cardiovascular drugs known to lower cardiovascular risk [22,23]: anti-platelet medication, statins, beta-blockers and renin-angiotensin-aldosterone system (RAAS) inhibitors was assessed at the moment of admission for PCI. Anti-platelet medication comprises aspirin and all types of P2Y_12_ inhibitors. RAAS inhibiting medication comprises all types of angiotensin-converting enzyme (ACE) inhibitors, angiotensin receptor blockers and aldosterone antagonists.

### SYNTAX scoring

The previously validated [14,24] SYNTAX score considers stenotic lesions reducing the luminal diameter >50% in vessels of >1.5mm.[13] In the total SYNTAX score, lesions are weighted depending on the anatomical position of the segment in which they occur. The more proximal in the coronary tree, the more points are assigned to a lesion. A SYNTAX score of >18 points corresponds to severe CAD and has been reported to be related to higher rates of adverse cardiac events.[24]

The SYNTAX score was measured by two independent observers (CG and AS), using SYNTAX score calculator [25] version 2.11. The observers were blinded to ethnicity and other patient characteristics. The two observers employed quantitative coronary angiography [26] (QCA) software (CAAS, Siemens) to measure the percentage of stenosis or the dimension of the vessel whenever they were uncertain about the angiographic significance of a lesion by visual estimation. QCA was performed for 95 cases in total (CG 67 cases, AS 47 cases). When the two observers were more than 5 SYNTAX points apart (60 cases), the case was discussed in order to reach consensus and QCA was performed to assess lesion significance. The average of the SYNTAX scores of each patient measured by both observers was used as a continuous outcome measure for the current analysis.

### All-cause mortality

The vital status (alive or deceased) of the Singaporean patients was extracted from state mortality registration and matched with individual patient data. In the Netherlands, follow-up was performed through annual patient follow-up questionnaires. When the patient did not respond, the general practitioner was contacted to obtain the patient’s vital status, which was subsequently added to the hospital registration. An extraction of the completed hospital registration was used for the current analysis.

### Statistical analysis

Continuous baseline variables are displayed as means with standard deviations (or confidence intervals for SYNTAX scores), and categorical variables are presented as percentages. The baseline characteristics were compared among the ethnic groups using ANOVA for continuous and chi-square testing for categorical data, respectively.

Baseline characteristics that differed significantly across the four ethnic groups or that were significantly associated with SYNTAX score on univariable analysis, were added to the multivariable model. Covariates in the multivariable model included: age, BMI, diabetes, dyslipidemia, smoking, previous PCI, previous ACS, peripheral arterial disease, use of platelet inhibitor, use of statin and use of beta blocker.

Inter-ethnic differences in crude SYNTAX scores were tested using ANOVA with Bonferroni post-hoc testing. Age-adjusted SYNTAX scores and SYNTAX scores adjusted for the covariates listed above were calculated with ANCOVA analysis. Differences in adjusted SYNTAX scores between the ethnic groups were tested using Tukey post-hoc testing.

Inter-ethnic differences in all-cause mortality were analyzed using Kaplan Meier analysis and Cox regression analysis with adjustment for age, sex, SYNTAX score and diabetes. Also, we tested for interaction between ethnicity and SYNTAX score for all-cause mortality in the multivariable Cox model.

The statistical analyses were performed using the R software package [27] for statistical computing, version 3.1.2. The data used for the purpose of this study are provided in S1 File.

## Results

### Patient characteristics

The characteristics of the stable CAD and STEMI patients are presented in Table 1.

**Table 1:** Baseline characteristics and SYNTAX scores of stable CAD and STEMI patients.

	Caucasian	Chinese	Indian	Malay	p-value
**Stable CAD patients**					
N	150	150	150	150	
Males (%)	83.3	81.3	77.3	78.0	0.52
Age (years, mean ± sd)	63.7±10.5	62.0±8.8	56.8±9.5	57.7±10.0	<0.001
BMI (kg/m^2^, mean ± sd)	28.0±4.4	26.5±4.8	27.4±5.1	29.2±4.9	<0.001
Diabetes (%)	23.5	36.0	58.0	52.7	<0.001
Hypertension (%)	64.0	78.0	69.3	71.3	0.06
Dyslipidemia (%)	57.2	77.3	78.0	75.3	<0.001
Current smoker (%)	24.6	28.8	40.0	47.0	0.013*
Ex smoker (%)	29.2	27.9	27.4	22.0	-
Non-smoker (%)	46.2	43.3	32.6	31.0	-
Previous PCI (%)	46.7	20.7	30.7	20.1	<0.001
Previous ACS (%)	33.3	17.4	26.8	18.0	0.002
CVA/TIA (%)	8.1	10.0	8.0	8.7	0.92
Peripheral arterial disease (%)	10.7	2.7	3.3	2.0	<0.001
Renal failure (%)	4.7	7.3	6.7	6.7	0.80
Anti platelet (%)	94.0	68.0	54.0	59.3	<0.001
Statin (%)	86.0	73.3	65.3	60.7	<0.001
Beta blocker (%)	74.7	40.0	47.3	52.7	<0.001
RAAS (%)	54.7	42.7	41.3	42.7	0.07
SYNTAX score (mean, 95% CI)	10.2 (9.1–11.3)	11.2 (10.2–12.1)	13.2 (12.0–14.4)	13.5 (12.4–14.6)	<0.001
Age adjusted SYNTAX score (mean, 95% CI)	10.1 (8.9–11.2)	11.1 (10.0–12.2)	13.3 (12.2–14.4)	13.6 (12.5–14.7)	<0.001
Fully adjusted SYNTAX score (mean, 95% CI)	9.4 (8.1–10.8)	11.8 (10.4–13.1)	13.4 (11.9–14.9)	13.4 (12.0–14.8)	<0.001
Median FU time (days)	575	575	1,243	1,169	
All-cause mortality (N)	9	6	8	14	
1-year mortality estimate (%)	4.7	4.0	2.0	4.1	0.43
**STEMI patients**					
N	100	100	100	100	
Males (%)	79.0	82.0	85.0	88.0	0.35
Age (years, mean ± sd)	61.1±10.6	60.0±12.6	52.6±11.1	54.5±10.4	<0.001
BMI (kg/m^2^, mean ± sd)	27.2±4.1	25.1±5.3	26.0±5.0	26.9±4.1	0.009
Diabetes (%)	13.0	35.0	46.0	41.0	<0.001
Hypertension (%)	39.4	54.0	42.0	47.0	0.17
Dyslipidemia (%)	32.3	57.0	66.0	64.6	<0.001
Current smoker (%)	44.7	53.2	69.1	79.5	<0.001*
Ex smoker (%)	22.3	14.3	10.3	8.4	-
Non-smoker (%)	33.0	32.5	20.6	12.0	-
Previous PCI (%)	7.0	7.0	12.0	10.0	0.53
Previous ACS (%)	6.0	10.0	11.1	10.0	0.62
CVA/TIA (%)	3.0	4.0	7.0	5.0	n/a
Peripheral arterial disease (%)	2.0	0.0	1.0	0.0	n/a
Renal failure (%)	0.0	0.0	3.0	2.0	n/a
Anti platelet (%)	42.0	9.0	11.0	9.0	<0.001
Statin (%)	27.0	21.0	23.0	21.0	0.71
Beta blocker (%)	27.0	13.0	10.0	11.0	0.002
RAAS (%)	25.0	18.0	18.0	11.0	0.08
SYNTAX score (mean, 95% CI)	14.0 (12.5–15.6)	18.5 (17.0–20.0)	16.1 (14.6–17.6)	18.6 (16.8–20.4)	<0.001
Age adjusted SYNTAX score (mean, 95% CI)	13.4 (11.8–15.0)	18.0 (16.5–19.6)	16.8 (15.3–18.4)	19.0 (17.4–20.5)	<0.001
Fully adjusted SYNTAX score (mean, 95% CI)	12.7 (10.9–14.6)	17.7 (159–19.5)	15.5 (13.5–17.4)	18.8 (17.1–20.6)	<0.001
Median FU time (days)	689	589	1,050	696	
All-cause mortality (N)	2	6	11	11	
1-year mortality estimate (%)	2	5	9	11	0.05

Figures represent percentages or means ± standard deviation. SYNTAX scores are presented as means with confidence intervals. Fully adjusted SYNTAX scores are adjusted for age, BMI, diabetes, dyslipidemia, smoking, previous PCI, previous ACS, peripheral arterial disease, use of platelet inhibitor, use of statin and use of beta blocker.

* p-value for difference across all smoking groups, across all ethnic groups. n/a: chi-square test results are not robust due to <5 observations in a group. SD = standard deviation, CI = confidence interval. Significance of differences was tested with ANOVA for continuous measures, ANCOVA for adjusted SYNTAX scores and chi-square testing for proportional measures.

Among the stable CAD patients (n = 600) Indian patients were the youngest (mean age 56.8±9.5 years) and Caucasians were the oldest (63.7±10.5, p-value for difference across all ethnic groups <0.001). There was no significant difference in the proportion of men versus women among the different ethnic groups. Diabetes was significantly more common among Indians and Malays (58.0% and 52.7%, respectively) than in Caucasians and Chinese (23.5% and 36.0%, respectively), p<0.001. Dyslipidemia was more prevalent in all Asian ethnic groups (Chinese 77.3%, Indians 78.0%, Malays 75.3%) than among Caucasians (57.2%, p<0.001), and smoking was more common among Indians and Malays (40.0% and 47.0% vs. 24.6% in Caucasians, p = 0.013). A history of previous PCI or ACS was significantly more common among Caucasians (p<0.001 and p = 0.002), as was the use of medications recommended by international guidelines for the treatment of CAD (p<0.001).

Among STEMI patients (n = 400), Indians and Malays were younger (52.6±11.1 and 54.5±10.4 years) than Chinese and Caucasians (60.0±12.6 and 61.1±10.6), p-value for difference across all ethnic groups <0.001). A high proportion of males was observed among Malays (88% as compared to 79%, 82% and 85% in Caucasians, Chinese and Indians, respectively). This difference was not statistically significant (p = 0.35). The prevalence of diabetes was two to three times higher among Chinese (35%), Indians (46%) and Malays (41%) than in Caucasians (13%), p-value overall <0.001. A history of PCI (7–12% across the ethnic groups) and history of ACS (6–11% across the ethnic groups) were equally common among the ethnic groups.

### SYNTAX scores in patients with stable CAD

**Fig 1:**
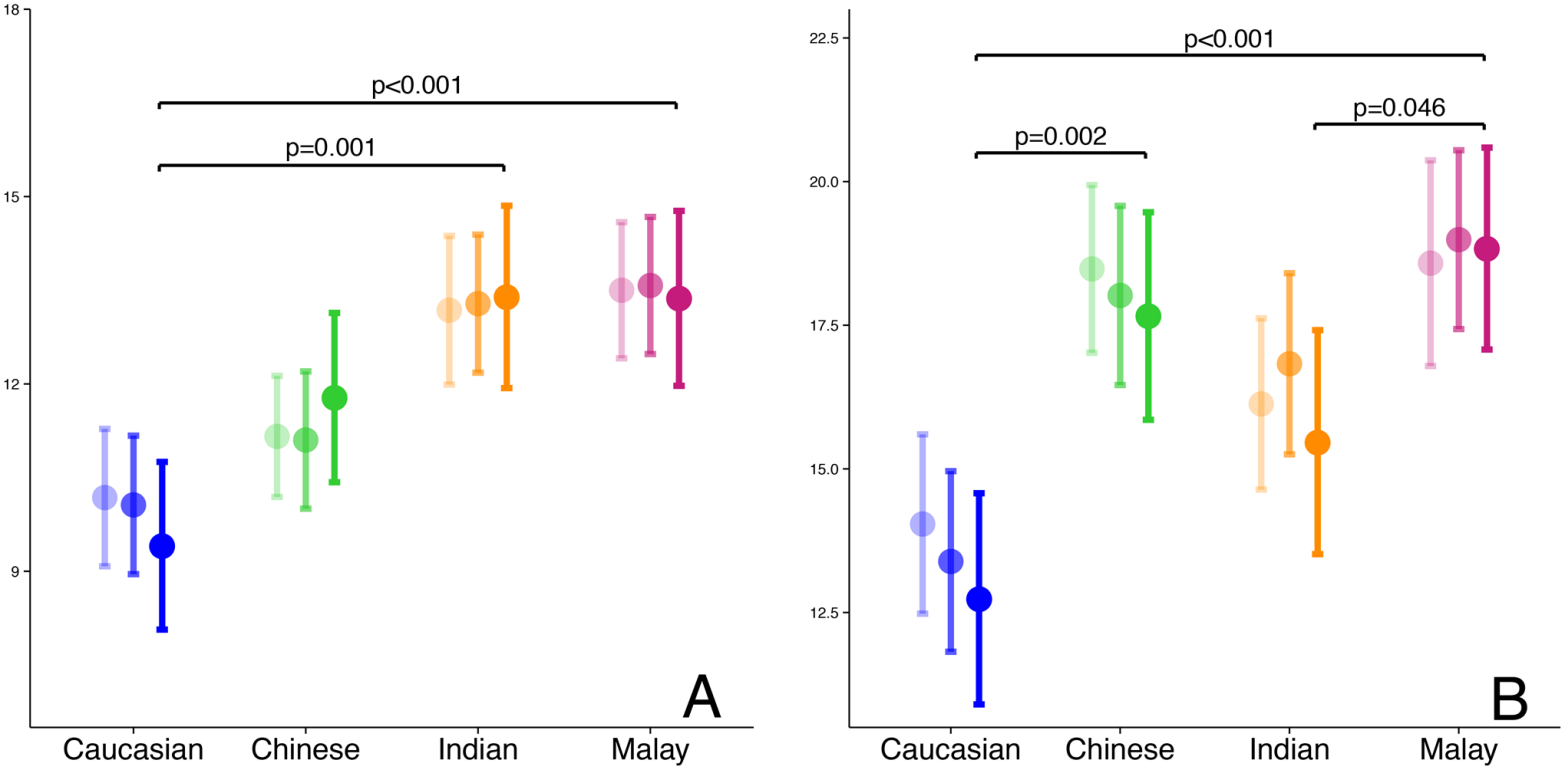
SYNTAX scores of stable CAD and STEMI patients, stratified by ethnicity. Panel A: SYNTAX scores of stable CAD patients (n = 150 per ethnic group). Panel B: SYNTAX scores of STEMI patients (n = 100 per ethnic group). Point estimates and error bars show the mean SYNTAX scores with 95% confidence intervals. Different transparencies present: crude mean SYNTAX scores (highly transparent), mean SYNTAX scores adjusted for age (lightly transparent) and multivariable adjusted mean SYNTAX scores (solid). P-values displayed in the figure are derived from multivariable (full model) ANCOVA, followed by Tukey post-hoc testing. The full model contains: age, body mass index, diabetes, dyslipidemia, smoking, previous PCI, previous acute coronary syndrome, peripheral arterial disease, platelet inhibitor, statin and beta-blocker use.

The crude and adjusted SYNTAX scores are depicted in Fig 1A. Crude SYNTAX scores (with 95% confidence intervals) were highest for Indians and Malays: 13.2 (12.0–14.4) and 13.5 (12.4–14.6), respectively. Age-adjusted mean SYNTAX scores for Caucasians, Chinese, Indians and Malays were 10.1 (8.9–11.2), 11.1 (10.0–12.2), 13.3 (12.2–14.4) and 13.6 (12.5–14.7). Even after multivariable adjustment, these ethnic differences in SYNTAX scores persisted: 9.4 (8.1–10.8), 11.8 (10.4–13.1), 13.4 (11.9–14.9) and 13.4 (12.0–14.8). Post-hoc testing revealed significantly lower SYNTAX scores in Caucasians when comparing with Indians (p = 0.001) or with Malays (p<0.001) in the multivariable model. Other comparisons did not reveal significant differences. The crude and adjusted SYNTAX scores are presented in Table 1.

### SYNTAX scores in STEMI patients

The crude and adjusted SYNTAX scores are depicted in Fig 1B. Crude SYNTAX scores for STEMI patients were highest in Chinese and Malays: 18.5 (17.0–20.0) and 18.6 (16.8–20.4), respectively, lower in Indians: 16.1 (14.6–17.6) and lowest in Caucasians: 14.0 (12.5–15.6). After adjustment for age, the scores of Caucasians and Chinese decreased to 13.4 (11.8–15.0) and 18.0 (16.5–19.6), respectively and the scores of Indians and Malays increased to 16.8 (15.3–18.4) and 19.0 (17.4–20.5). SYNTAX scores, fully adjusted for differences in baseline characteristics were: Malays 18.8 (17.1–20.6), Chinese 17.7 (15.9–19.5), Indians 15.5 (13.5–17.4) and Caucasians 12.7 (10.9–14.6).

Post-hoc testing revealed that the fully adjusted SYNTAX scores of Caucasians differed from the scores of Chinese and Malays (p = 0.002 and p<0.001). And the scores of Indians differed from the scores of Malays (p = 0.046). Other post-hoc comparisons between ethnic groups did not yield a significant difference.

### Mortality

**Fig 2:**
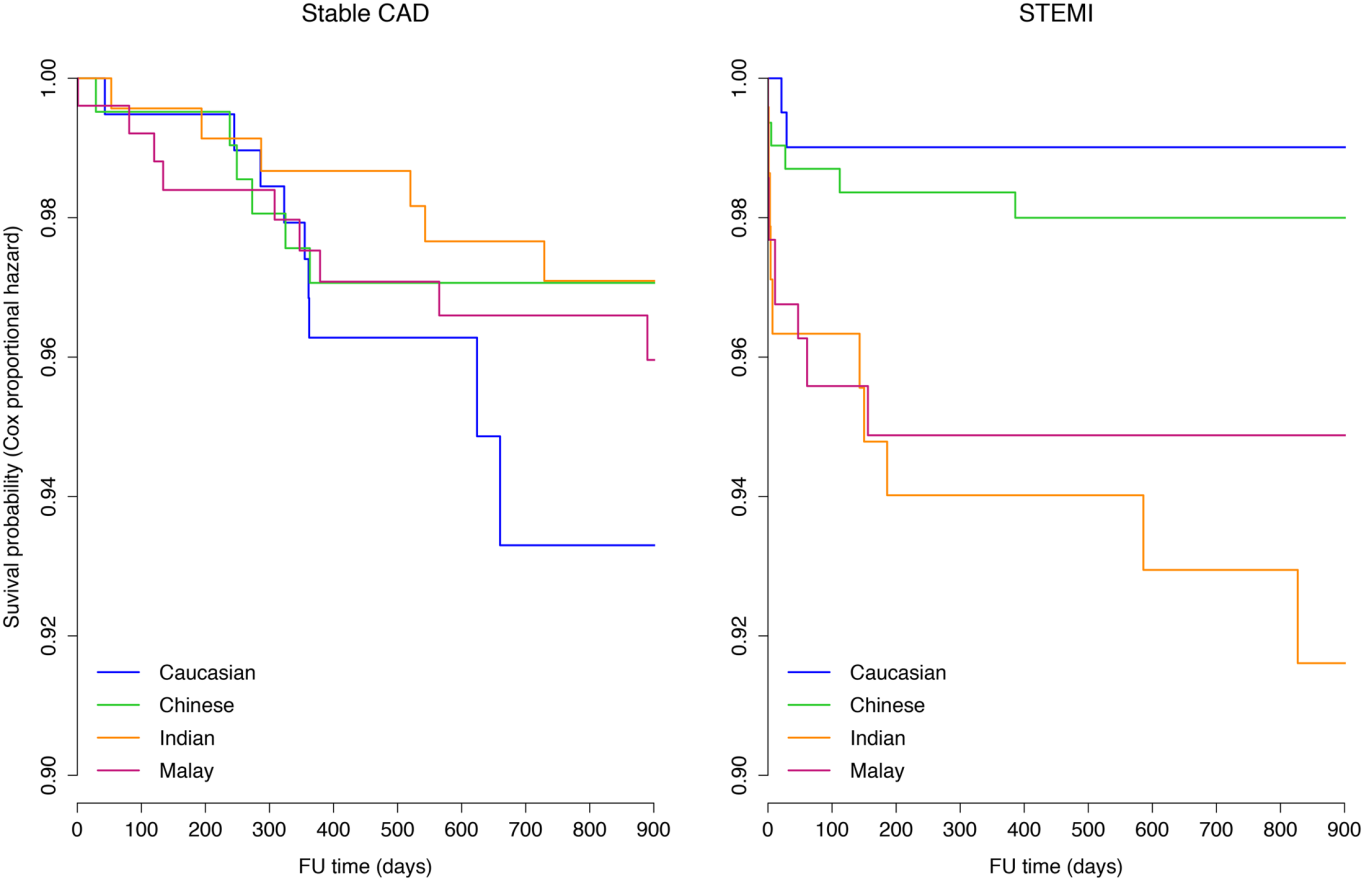
Cox regression survival curves for up to 900 days of follow-up stratified by ethnicity. Cox regression survival curves for up to 900 days of follow-up stratified by ethnicity. The survival curves are adjusted for age, sex, SYNTAX score and diabetes. The left panel displays the stable CAD patients, the right panel the STEMI patients. No significant ethnic differences were found among stable CAD patients. Among the STEMI patients, mortality was significantly higher in Malays (HR 5.8) and Indians (HR 7.2) as compared to Caucasians.

In patients with stable CAD, unadjusted all-cause mortality rates did not significantly differ between the four ethnic groups (Fig 2, left panel). Among STEMI patients, however, all-cause mortality was highest in Malays, reaching 11% at one year (p = 0.053 for difference among the four ethnic groups).

Among stable CAD patients, the multivariable Cox regression model including age, sex, SYNTAX score and diabetes as covariates, neither SYNTAX score nor ethnicity independently predicted all-cause mortality; whilst diabetes (HR 3.4, 1.6 to 7.3, p = 0.001) and age (HR 1.6, 1.1 to 2.2, p = 0.005) remained independent predictors of all-cause mortality. There was no interaction between SYNTAX score and ethnicity in the regression with all-cause mortality as the outcome.

In STEMI patients (Fig 2, right panel), however, Indian and Malay ethnicity as compared to Caucasian ethnicity appeared to be significant independent predictors of all-cause mortality (HR 7.2, 1.5 to 34.7, p = 0.01 and HR 5.8, 1.2 to 27.2, p = 0.03, respectively) when adjusted for age, sex and diabetes. The SYNTAX score was an independent predictor of all-cause mortality (HR 2.5, 1.7 to 3.8, p<0.001 for every 10 to point increase) among STEMI patients, irrespective of ethnicity. There was no significant interaction between ethnicity and SYNTAX score for all-cause mortality in STEMI patients, indicating an equal predictive value of SYNTAX score across the ethnic groups.

## Discussion

In this comparison of CAD severity among four of the world’s most populous ethnic groups using a well-validated quantitative score of angiographic CAD severity (SYNTAX score), we observed clear ethnic differences in the severity of angiographic CAD among stable CAD and STEMI patients undergoing PCI. Indians and Malays with stable CAD had higher SYNTAX scores (reflecting more severe CAD) than Caucasians and Chinese. These differences were not attenuated when adjusting for confounders, indicating that ethnic differences in the prevalence of known traditional risk factors could not entirely explain the observed differences in CAD severity as quantified by the SYNTAX score.

Indian and Malay ethnicity (in comparison to Caucasian ethnicity) were significantly associated with higher all-cause mortality in STEMI patients undergoing PCI. This remained so, after adjusting for age, SYNTAX score and diabetes. Among stable CAD patients undergoing PCI, no significant differences in mortality rates were found among the ethnic groups. No interactions were found between ethnicity and SYNTAX score for stable CAD and STEMI patients in the prediction of all-cause mortality, indicating that SYNTAX score has similar predictive properties among the ethnic groups.

### Ethnicity and the severity of CAD

The association of Chinese ethnicity with higher SYNTAX scores among STEMI patients is intriguing, as Chinese ethnicity is often associated with a more favorable risk factor profile (e.g. lower cholesterol levels [11]) and lower cardiovascular mortality (2-year adjusted cardiovascular mortality rate of 1.8% vs. 4.5% among Whites [28]) than other ethnic groups. This pattern is also observed in Singapore.[29] A lower angiographic burden of CAD has been described by Jiang et al.[12] for Chinese (China) as compared to Caucasians (Australia), in a population with suspected CAD, in whom subsequent treatment was not specified. In contrast, a comparable burden of CAD between Chinese (China) and Caucasians (Germany) was reported by Zheng et al.[30] They described patients with myocardial infarction or chest pain with a significant lesion upon coronary angiography. But, only part of this study population required revascularization, with more revascularization required in the Caucasian group than in the Chinese group, implying already a more diseased patient group in Caucasians than in Chinese. Our results could imply that at the point of developing symptoms of STEMI, Chinese patients have more advanced CAD than their Caucasian counterparts, as evidenced by significantly higher SYNTAX scores in the STEMI group (p = 0.001).

Indians (or South Asians) have repeatedly been described as a high risk group for early onset and severe CAD.[31] For example, Vallapuri et al.[32] described a coronary angiography cohort of patients with significant CAD, and found a higher prevalence of triple vessel disease in Asian Indians (India) as compared to Caucasians (USA) with an associated higher Gensini [33] score. Compared with Caucasians, we observed high SYNTAX scores only in Indian patients with stable CAD but not in Indians presenting with STEMI.

A key finding in our study is that patients of Malay descent have more severe CAD as compared to the other ethnic groups, regardless of whether they suffer from stable CAD or STEMI. Higher all-cause mortality in Malay STEMI patients has been described [34] and our finding of greater CAD severity in patients of Malay descent may in part explain their poorer outcomes.

### Ethnicity, CAD severity and mortality

For Indians, all-cause mortality from acute coronary syndromes has been described to be lower as compared to Caucasians [35], which is in contrast with our results. However, in the study by Zaman et al.[35] only 36% of the study population was comprised of STEMI patients, and an even lower percentage received primary PCI. Within the STEMI patients of their study, the mortality in Indians was higher compared to the Caucasians, which is in agreement with our findings.

Chinese are known to have lower CAD-attributable mortality rates than Caucasians in the general population.[36] However, the literature is inconclusive about mortality among Chinese CAD patients. Gasevic et al. [37] and Khan et al. [38] reported that after myocardial infarction Chinese are at higher risk of dying in the first 30 days after PCI than Caucasians. Long-term all-cause mortality did not differ significantly between Chinese and Caucasians in the analysis by Khan et al. [38] In contrast, Qian et al. [39] reported that Asian Americans (of whom the largest proportion is Chinese [40]) show better survival after myocardial infarction than Caucasians. In our current study with a median follow-up duration of 709 days, we did not find a difference between survival in Chinese and Caucasian patients. Due to the chance of a type II error and possible under-powering of the current study, we cannot exclude the possibility of an actual difference in all-cause mortality between Chinese and Caucasians, however our data indicate no striking numerical differences in mortality between Chinese and Caucasians.

To date, no comparison between Caucasians and Malays has been reported in the literature. However, ten years ago, Mak et al. [34] reported Malays to have a poorer prognosis after myocardial infarction than Chinese and Indians in Singapore (HR 1.26 as compared to Chinese ethnicity), supporting the results of high SYNTAX scores and high rates of all-cause mortality among Malays in the current study.

Klomp et al. [3] reported better survival in Asian patients undergoing PCI, mostly of Malay descent, than in Western Europeans undergoing PCI. This important earlier study, however, encompassed all indications for PCI, and elective PCI was significantly more common in the Asian ethnic group than in the Western European group; while survival analysis was not adjusted for PCI indication.

In our study we find differences in all-cause mortality among STEMI patients, but not among stable patients. The STEMI patient group is a very homogeneous patient group with a clear phenotype. The stable CAD patient group, on the other hand, might encompass a wider range of patients, thereby masking a possible effect of ethnicity by subtle differences in stable indications for angiography among the ethnic groups.

### Possible mechanisms of inter-ethnic differences in CAD severity

The association of ethnicity with SYNTAX score is likely to be of a multifactorial origin. It is conceivable that there are cultural lifestyle or country-dependent primary prevention strategies differences among the ethnic groups, which are not completely reflected by the cardiovascular risk factor profile. Reports suggest that CAD outcomes are strongly linked to a country’s economic prosperity.[41] Remarkably, Singapore has a per capita GDP of US$ 55,182, higher than the Dutch GDP of US$ 50,793 (data for 2010–2014 from data.worldbank.org).

On the other hand, there might be true genetic [42,43] and biological differences among the ethnic groups of which only the tip of the iceberg is known. For example, a stronger association of cholesterol and diabetes with carotid intima-media thickness has been shown for Asian Indians as compared to Caucasians, suggesting higher susceptibility of the arterial vasculature to cholesterol and glucose levels.[44] In our study we were only able to take the absence or presence of dyslipidemia and diabetes into account, and not the actual biomarker levels reporting on these diseases. It is possible that the actual cholesterol levels or glycated hemoglobin levels associate with SYNTAX score in different ways across the ethnic groups and thus explain part of the association of ethnicity with SYNTAX score that we find.

Unraveling the underlying causes of these ethnic differences in CAD severity is critical when tackling the epidemic of CAD in Asia. When biological [45] and epidemiological connections between ethnicity and CAD become more delineated, ethnicity-specific prevention and treatment strategies can be developed to improve survival in the ethnic groups with poorest prognoses.

### Limitations

In this study we were unable to correct for lifestyle factors beyond those that were described in the baseline table. It is possible that dietary, socioeconomic factors obscure the true association of ethnicity with the angiographic severity of CAD into some extent. Furthermore, lifestyle and medication adherence may also explain differences in mortality following PCI.

By specifically including PCI patients we created a homogenous patient group; however the choice for PCI might have been steered by patient preferences (refusing CABG). We were unable to take patient preferences for revascularization strategy into account and it is possible that patient preferences differ among ethnic groups.

While the SYNTAX score is the most widely used scoring method to quantify the angiographic severity of CAD, it does have limitations. For example, SYNTAX score does not distinguish between a lesion of 50% and a lesion of 99% luminal stenosis.[13] Plus, the assessment of significance of a lesion is performed by visual estimation and thus observer-dependent (although inter-observer correlation in our study was high, r = 0.95).

Due to a limitation in statistical power we could not extend our multivariable survival analyses with more covariates.

## Conclusion

In both stable CAD and STEMI patients undergoing PCI, SYNTAX scores were higher in Malays and Indians as compared to Caucasians, also after adjustment for relevant covariates. After STEMI, mortality was significantly higher among Indian and Malay patients as compared to Caucasian patients, even after multivariable adjustment including for SYNTAX score.

Future research should focus on understanding underlying intrinsic biological and environmental factors accounting for these differences in CAD severity and case-fatality, so as to identify better targets for early intervention.

## Supporting Information

S1 File Data. Data used for the purpose of this study.
(CSV)
